# Detection of local recurrent prostate cancer after radical prostatectomy in terms of salvage radiotherapy using dynamic contrast enhanced-MRI without endorectal coil

**DOI:** 10.1186/1748-717X-7-185

**Published:** 2012-10-31

**Authors:** Hans Christian Rischke, Arnd O Schäfer, Ursula Nestle, Natalja Volegova-Neher, Karl Henne, Matthias R Benz, Wolfgang Schultze-Seemann, Mathias Langer, Anca L Grosu

**Affiliations:** 1 Department of Radiation Oncology, University of Freiburg, Robert Koch Str. 3, Freiburg, 79106, Germany; 2 Department of Diagnostic Radiology, University of Freiburg, Freiburg, Germany; 3 Department of Molecular and Medical Pharmacology, University of California in Los Angeles, Los Angeles, USA; 4 Department of Urology, University of Freiburg, Freiburg, Germany

**Keywords:** Prostate cancer, PSA recurrence, Salvage radiotherapy, Dynamic contrast enhanced MRI, Gross tumor volume

## Abstract

**Purpose:**

To evaluate the value of dynamic contrast enhanced Magnetic Resonance Imaging (DCE-MRI) without endorectal coil (EC) in the detection of local recurrent prostate cancer (PC) after radical prostatectomy (RP).

**Material and methods:**

Thirty-three patients with recurrent PC underwent DCE-MRI without EC before salvage radiotherapy (RT). At median 15 (mean 16±4.9, range 12–27) months after completion of RT all patients showed complete biochemical response. Additional follow up post RT DCE-MRI scans were available. Prostate specific antigen (PSA) levels at the time of imaging were correlated to the imaging findings.

**Results:**

In 22/33 patients (67%) early contrast enhancing nodules were detected in the post-prostatectomy fossa on pre-RT DCE-MRI images. The average pre-RT PSA level of the 22 patients with positive pre-RT DCE-MRI findings was significantly higher (mean, 0.74±0.64 ng/mL) compared to the pre-RT PSA level of the 11 patients with negative pre-RT DCE-MRI (mean, 0.24±0.13 ng/mL) (p<0.001). All post-RT DCE-MRI images showed complete resolution of initial suspicious lesions. A pre-RT PSA cut-off value of ≥0.54 ng/ml readily predicted a positive DCE-MRI finding.

**Conclusions:**

This is the first study that shows that DCE-MRI without EC can detect local recurrent PC with an estimated accuracy of 83% at low PSA levels. All false negative DCE-MRI scans were detected using a PSA cut-off of ≥0.54 ng/mL.

## Background

Salvage radiotherapy (RT) of the prostate fossa is considered the standard therapeutic intervention that offers a potential of cure for patients with post-radical prostatectomy (RP) local recurrence [1]. Serum prostate specific antigen (PSA) kinetic is the most accurate and early index for detecting prostate cancer (PC) recurrence after RP [2,3]. Dependent on initial tumor stage, PSA level, Gleason-score and resection status, 10 to 53% of patients develop a biochemical relapse following RP [4].

Local PC recurrence can most accurately be detected on Magnetic Resonance Imaging (MRI) [5,6]. The sensitivity and specificity of this imaging test was further improved by the introduction of dynamic contrast enhanced (DCE)-MRI with endorectal coil (EC) [7,8]. DCE-MRI visualizes tumor neo-vascularisation, which is typically found in PC, showing rapid enhancement in the first 90 seconds after intravenous administration of gadolinium contrast agent [9-11].

Dose escalation in salvage radiation therapy has been shown to improve PSA relapse free rates [12-14]. However, higher radiation dose delivery to the prostate fossa, the target volume in clinical practice, increases the risk of acute and chronic side effects to adjacent organs at risk, predominantly rectal wall, bladder, and bowel loops located in the pelvis [15].

A recently published regression meta-analysis of twenty five published reports about the outcome of salvage RT comprised a total of 3828 patients [16]. In this study Ohri et al. identified increased salvage RT dose and decreased pre-salvage RT PSA level as independent predictors of improved 5-year biochemical free survival. Salvage RT dose was also identified as an independent predictor of both late gastrointestinal and late genitourinary toxicity. They generated tumor control probabilities (TCP) and normal tissue complication probabilities (NTCP) models that can be used to predict rates of disease control and severe late toxicity. For example with a pre-salvage RT PSA-level of 0.4 ng/mL an approximately 50% chance of 5-year biochemical free survival can be achieved with a salvage RT dose of 60 Gy. Severe late toxicity rates with this dose are on the order of 1%. If the PSA level before salvage RT is 1.0 ng/mL, than dose of approximately 70 Gy may be required to achieve the same probability of disease control, but severe late toxicity rates at that dose level may reach 10%, if those patients will be treated with conventional RT-technique. The model by Ohri et al. demonstrated that the gain from dose escalation is greater when PSA is high (that means PSA of 1.5 ng/mL).

MRI with endorectal coil is superior over computer tomography (CT) in detecting local recurrent PC. However, MRI with endorectal coil causes a distortion of local anatomy and therefore does not allow image fusion with RT-planning CT to define a gross tumor volume (GTV) for targeted dose escalation.

An Imaging modality to identify and correctly localize local PC recurrence without induced distortion of the local anatomy in the small pelvis is therefore strongly desirable and may offer the opportunity to reduce acute and late toxicities.

Therefore, the aims of this retrospective study were threefold. First, we determined the accuracy of DCE-MRI without endorectal coil in the detection of local recurrent PC at low PSA levels. Secondly, we assessed the predictability of a false negative DCE-MRI without EC. Thirdly, we correlated enhancing tumor nodule size to pre-RT PSA-level.

## Patients and methods

### Inclusion criteria

The database of the University Hospital of Freiburg was retrospectively analysed for post-RP patients that were treated by salvage RT of the whole fossa prostatica for local recurrent PC between January 2007 and January 2010. Of these patients, only those who showed complete biochemical response ≥ 12 months after completion of salvage RT, defined by a PSA below detection level, and those who underwent a DCE-MRI without EC of the pelvis before the initiation of salvage RT were included in this study. Additionally metastatic disease was ruled out using Choline-PET or Choline-PET/CT imaging in all patients. Patients who underwent anti-androgen therapy before or after salvage RT were excluded from this analysis. Follow up DCE-MRI without EC had to be performed ≥ 12 months after completion of salvage RT and was used as internal reference to specify whether contrast enhancing tumor lesions in the prostate fossa show complete response in accordance to complete biochemical response.

For this retrospective study, the University of Freiburg Institutional Review Board waived the consent requirements.

### DCE-MRI technique

DCE-MRI scans were acquired on 1.5-Tesla systems (Magnetom Avanto, Espree or Symphony, Siemens Medical Solutions, Erlangen, Germany), each equipped with surface phased array (Body Matrix, Siemens Medical Solutions). MRI acquisition parameters differed between scanners. Imaging of the post-prostatectomy fossa was performed by acquiring turbo spin echo (TSE) T2-weighted sequences in the axial, sagittal and coronal planes (repetition time [TR], 9200–9500 ms; echo time [TE], 119–128 ms; flip angle 150–160; field of view 150–300 mm; thickness 3 mm; section gap 0; matrix, 192 x 192 to 512 x 512).

An axial turbo spin echo (TSE) T1-weighted series of the whole pelvis was then obtained with the following parameters: repetition time [TR], 798 ms; echo time [TE], 9,8 ms; flip angle 150; field of view 380 mm; thickness 3 mm; section gap 0; matrix, 384 x 288. The last series performed was a 3D, fast low-angle shot (FLASH), T1-weighted spoiled gradient-echo sequence in axial plane (TR, 4.3 – 5.41 ms; TE, 1.82-2.39 ms; flip angle 10–15, field of view 380 mm, thickness 2 mm; section gap 0; matrix, 384 x 264 – 384 x 288) to perform 4–10 measurements in rapid succession, immediately following completion of an intravenous bolus injection of 0.15 ml/kg gadopentetate dimeglumine (Multihance, Bracco) using a power injector (Medtron) at 2 ml/s followed by a 30 ml saline flush. Therefore time resolution varied between 20 and 40 seconds.

### Image analysis

A board certified radiologist with 8 years of experience in urogenital MRI imaging (HCR) who was blinded to clinical and biochemical information analysed all pre- and post-RT DCE-MRI scans. The lesion location was recorded and the perpendicular diameter was calculated as follows: 4/6π xyz, while x, y and z are perpendicular diameters.

Pre- and post-RT DCE-MRI images of the prostatic bed were analysed according to following classifications:

True positive DCE-MRI: Suspicious early contrast enhancing lesion (visual determined peak enhancement within 90 seconds post injection) in the post-prostatectomy fossa on pre-RT DCE-MRI scan and elevated PSA level (≥0.08 ng/mL).

False positive DCE-MRI: Suspicious early contrast enhancing lesion (visual peak enhancement within 90 seconds post injection) in the post-prostatectomy fossa on pre-RT DCE-MRI scan and elevated PSA level (≥0.08 ng/mL), that shows no significant morphologic changes on post-RT DCE-MRI while PSA remission is detected.

False negative DCE-MRI: Elevated PSA level (≥0.08 ng/mL) but absence of a suspicious contrast enhancing lesion in the post-prostatectomy fossa on pre RT DCE MRI scan.

True negative DCE-MRI: Absence of a suspicious contrast enhancing lesion in the post-prostatectomy fossa on DCE-MRI scan, and PSA below detection level. Because all 33 patients showed a complete biochemical response, all post RT-DCE-MRI scans were used as an internal negative control in this setting. This is based on the fact that radiotherapy is a highly effective treatment and on the consideration that RT has the potential to entirely degrade tumor nodules including their neo-vascularization leading to non-enhancing post-RT-fibrosis similar to non-enhancing post-surgical fibrosis in the post prostatectomy fossa [8,17].

### Treatment characteristics

All patients received salvage RT of the whole fossa prostatica with 63.0 to 70.2 Gy (mean 68.1, median 68.2 Gy), 5 × 1.8 Gy/week. Dose escalation to 70.2 Gy was performed, when both diagnostic methods, DCE-MRI as well as Choline-PET detected a finding suspicious of local recurrence in the fossa prostatica.

### Statistical analysis

Quantitative data are presented as median, mean ± SD and range. The Mann–Whitney test was used for unpaired comparisons between quantitative parameters. The Spearman rank test was used for correlations between PSA levels and tumor volume/diameters. P values <0.05 were considered statistically significant. Statistical analyses were performed using SPSS^©^ software for Windows^©^ (version 14.0, SPSS Inc., Chicago, USA) and JMP^©^ software for Windows^©^ (release 5.0.1.2).

## Results

### Patient characteristics

Between January 2007 and January 2010, 54 patients underwent post-RP salvage RT for local recurrent prostate cancer in our institution. Thirty-three of the 54 consecutive patients (61%) met the above listed inclusion/exclusion criteria.

The time interval between RP and PSA recurrence averaged 46±41.6 months (median, 35 months: range, 4–204 months). Salvage RT was initiated 2.7±2.2 weeks (median, 2 weeks; range, 1–12 weeks) after the pre-RT DCE-MRI. The post-RT DCE-MRI was performed at median 15 months (mean 16±4.9, range, 12–27 months) following RT.

### PSA levels

The PSA level averaged 0.57±0.58 ng/ml (median, 0.34 ng/mL; range, 0.08-2.38 ng/mL) at the time of local recurrence. All patients exhibited a complete biochemical response following RT, PSA was then below detection level (detection cut off level varied between different laboratory tests, mean, 0.02±0.03 ng/mL; median, 0.00 ng/mL; range 0.00 - 0.07 ng/mL) (Table 1).

**Table 1:** Patient characteristics (n=33)

**Characteristics**	
Age (years) at PSA recurrence	
Median (range)	69 (55 – 80)
T-stage at surgery	
pT4	3 (9)
pT3a/b	11 (33)
pT2a-c	19 (58)
Gleason-Score at surgery	
6	1 (3)
7	24 (73)
8	6 (18)
9	2 (6)
Surgical margins	
Positive	11 (33)
Negative	15 (46)
Unknown	7 (21)
PSA level prior to surgery (ng/mL)	
Median	9.8
Mean±SD	11.4±6.0
Range	2.9-25
PSA level at recurrence (ng/mL)	
Median	0.34
Mean±SD	0.57±0.58
Range	0.08-2.38
PSA level at follow-up-MRI (ng/mL)	
Median	0.00
Mean±SD	0.02±0.03
Range	0.00-0.07

### DCE-MRI findings

In 22 of the 33 patients (67%) a total of 24 suspicious early contrast enhancing nodules were detected, located in the post-prostatectomy fossa on pre-RT DCE-MRI images. The lesion’s characteristics are summarized in Table 2. Lesions exhibited a complete morphologic response along with a complete biochemical response post-RT, and were therefore classified as true positive.

**Table 2:** Characteristics of lesions classified as true positive

**Characteristics**	**n (%)**
Number of suspicious lesions per patient	
unifocal	20 (91)
bifocal	2 (9)
Location of suspicious lesions*	
perianastomotic	19
retrovesical	4
other (peribulbic)	1
Shape of suspicious lesions	
round	18
planelike, elliptic	4
irregular	2
Diameter of suspicious lesions (mm)	
Median	10
Mean	11±/4.4
Range	5-20
Volume of suspicious lesions (mL)	
Median	0.2
Mean	0.4±0.4
Range	0.1-1.5

*Two patients had bifocal lesions, one of them in two different locations therefore the numbers add up to 24.

All but two lesions were located between the 1 and 10 o’clock position in relation to the vesicourethral anastomosis, most frequently around the posterior and lateral aspects. One lesion was located between the 10 and 12 o’clock, and another one between the 11 and 1 o’clock position, respectively.

The maximum pre-RT tumor diameter in the 24 lesions averaged 1.1±0.4 cm (median, 1.0 cm; range, 0.5 – 2.0 cm). The average tumor volume measured 0.4±0.4 mL (median 0.2 mL; range, 0.1 – 1.5 mL) (Table 2).

**Figure 1:**
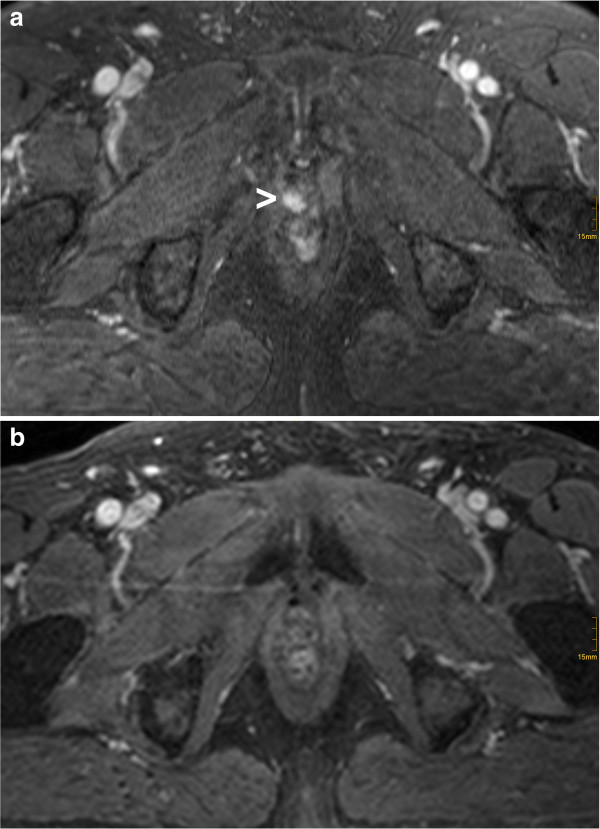
**Example of a patient classified as true positive.** A contrast enhancing lesion was detected on pre-RT DCE-MRI (**a**) (arrowhead), with completely morphologic response on the post-RT DCE-MRI (**b**).

Figure 1a and b show a pre-RT and post-RT DCE-MRI without EC of a patient classified as true positive.

Eleven of the 33 patients (33%) did not exhibit a suspicious lesion on the pre-RT DCE-MRI scans, but elevated pre-RT PSA levels (mean 0.24±0.13 ng/mL; median, 0.22 ng/mL; range, 0.08 - 0.53 ng/mL), with post-RT showing decrease below detection levels (complete PSA remission). Therefore, 11 patients were classified as false negative.

All post-RT DCE-MRI showed absence of suspicious contrast enhancing lesions together with complete PSA remission post-RT. Therefore, the post-RT DCE-MRI findings were used as a negative control to calculate the specificity as well as the positive (PPV), the negative predictive value (NPV), and the accuracy of DCE-MRI without EC. The sensitivity and specificity of DCE-MRI without endorectal coil in detecting local recurrent PC was 67% and 100%, respectively. The PPV, NPV, and the accuracy was 100%, 75% and 83%, respectively.

### Correlation between tumor volume/size and PSA level

**Figure 2:**
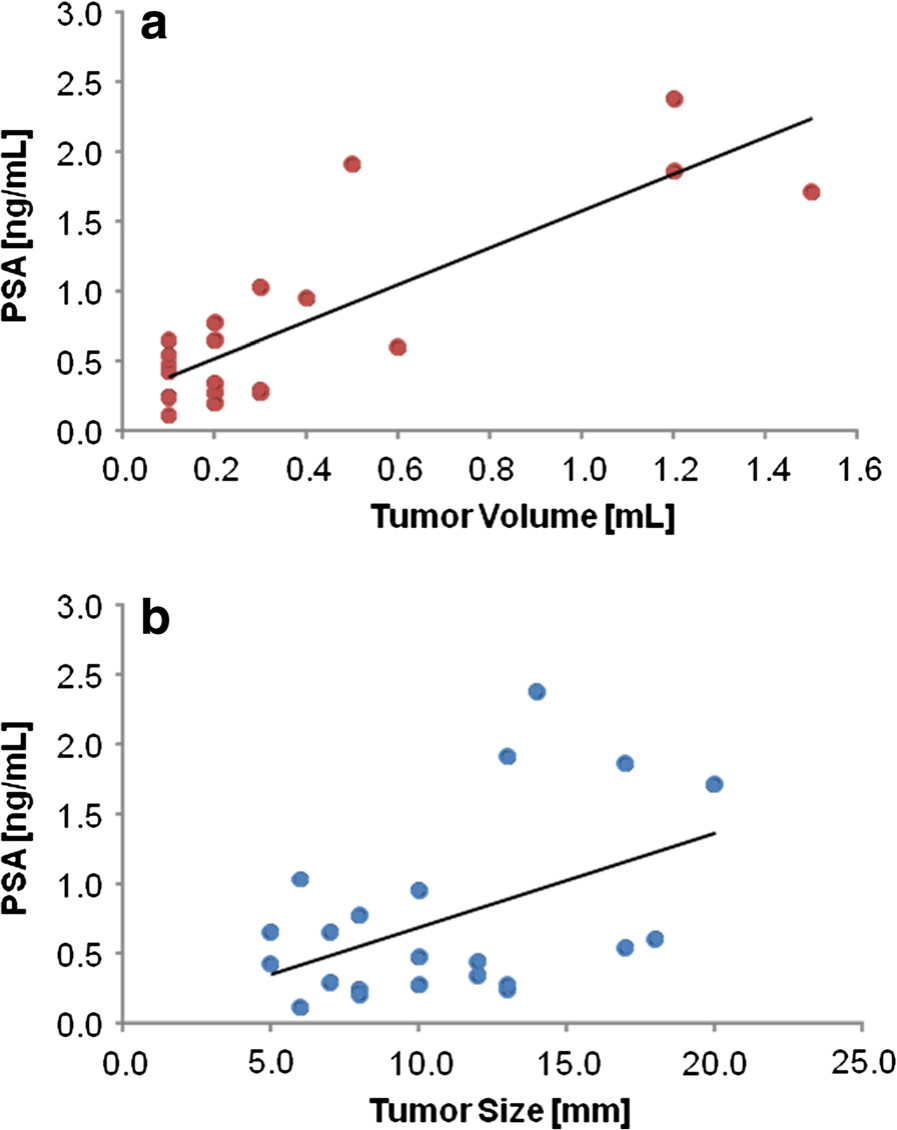
**(a) Correlation between tumor volume and PSA (Spearman correlation coefficient, 0.83; p<0.0001).** (**b**) Correlation between tumor size and PSA (Spearman correlation coefficient, 0.47; p=0.03).

The pre-RT tumor diameters and tumor volumes of the 22 patients with positive pre-RT DCE-MRI findings were significantly correlated to pre-RT PSA levels (Spearman rank correlation coefficient for correlation between tumor volume and PSA level = 0.83, p<0.001 (Figure 2a); Spearman rank correlation coefficient for correlation between tumor diameter and PSA level = 0.47, p=0.03 (Figure 2b)).

### Predictability of a positive DCE-MRI finding

**Figure 3:**
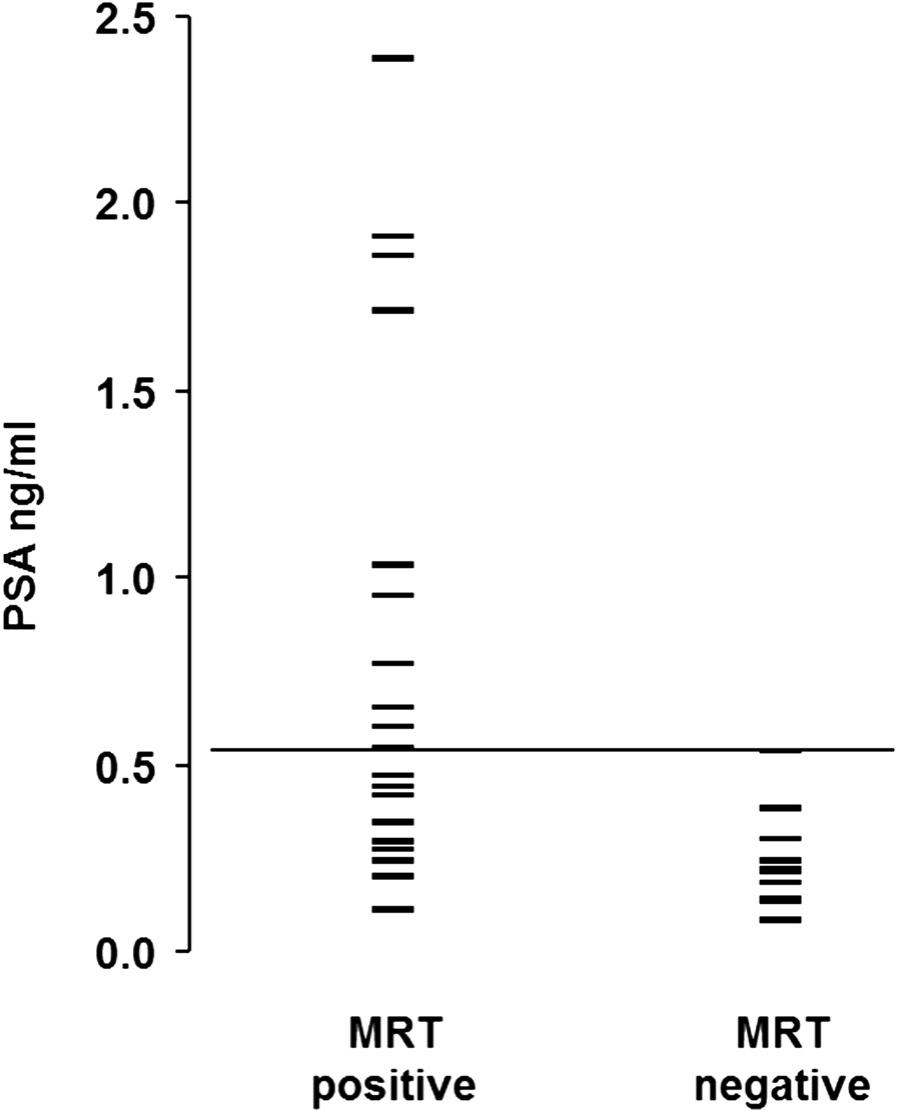
Scatter plot of pre-RT PSA levels in patients classified as true positive and false negative.

The average pre-RT PSA levels of the 22 patients with positive pre-RT DCE-MRI findings were significantly higher (mean, 0.74±0.64 ng/mL; median, 0.51 ng/mL, range, 0.11-2.38) compared to the pre-RT PSA levels of the 11 patients with negative pre-RT DCE-MRI (mean, 0.24±0.13 ng/mL; median, 0.22 ng/mL, range, 0.08-0.53) (p<0.001) (Figure 3). A pre-RT PSA cut-off value of ≥0.54 ng/ml readily predicted a positive DCE-MRI finding (Figure 3).

### Characterization of a common DCE-MRI pattern

**Figure 4:**
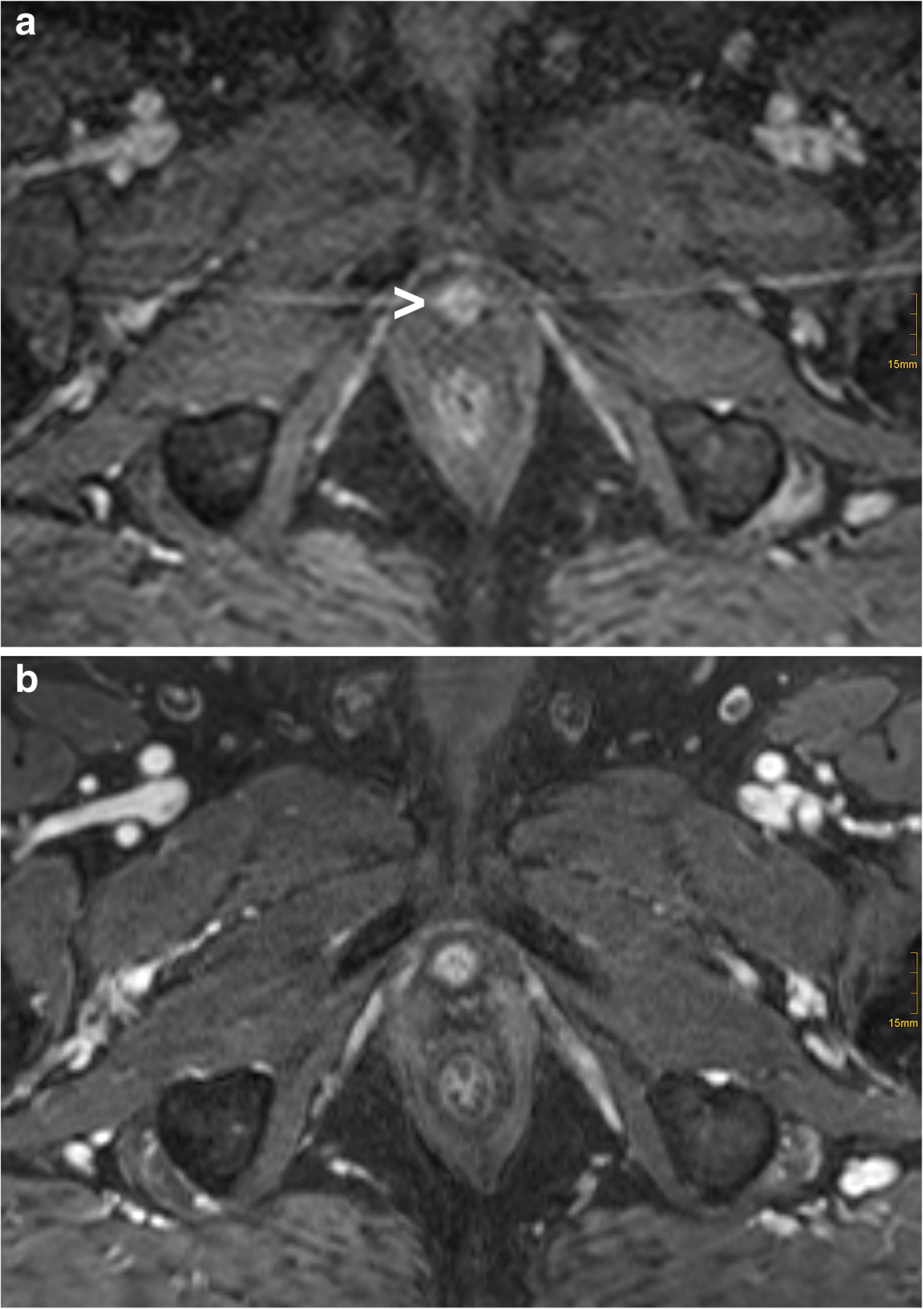
**Contrast enhancement at the anterior aspect of the proximal urethra immediately below the vesicourethral anastomosis on pre-RT DCE-MRI which was not associated to the patient’s recurrent prostate cancer (a) (arrowhead).** Post-RT DCE-MRI demonstrating no obvious change of this structure following RT (**b**).

We observed in 27 of the 33 patients (82%) an unequivocal contrast enhancement in the diaphragma urogenitale at the anterior aspect of the proximal urethra immediately below the vesicourethral anastomosis, with no obvious change on post-RT DCE-MRI demonstrating no obvious change following RT (Figure 4a and b). In 6 of the 33 patients (18%) no enhancement of the proximal urethra was evident. The periurethral enhancement was located at midline with a lobulated or circular aspect of symmetrical shape. The diameter averaged 1.0±0.3 cm (median, 1.0 cm; range, 0.5 – 1.9 cm).

## Discussion

To the best of our knowledge this is the first study that describes the detectability of recurrent PC using DCE-MRI without EC in the setting of very low PSA-levels.

DCE-MRI without EC detected recurrent PC with an accuracy of 83%. The pre-RT tumor volumes of the 22 patients with positive pre-RT DCE-MRI findings showed high correlation to pre-RT PSA levels. Our results also indicate a correlation between lesion detectability and increasing PSA-level. A PSA cut-off of ≥0.54 ng/mL detected all lesions positive on DCE-MRI as true positive. We also alert radiation oncologists to acknowledge a commonly observed unequivocal contrast enhancement at the anterior aspect of the proximal urethra immediately below the vesicourethral anastomosis, which represents physiological contrast enhancement.

Modern three-dimensional RT-planning enables the delineation of a GTV for targeted dose escalation on RT-planning CT scans. However, recurrent PC is usually missed on RT-planning CT due to tumor densities equal to the surrounding tissues. Therefore, local recurrent PC is managed so far by delivering RT to the entire fossa prostatica, knowing that local recurrences tend to be located around the vesicourethral anastomosis, but usually without having precise information where the recurrence is located and furthermore without having the possibility to fuse a precise image-based information with the RT-planning CT [15].

DCE-MRI in combination with EC was recently validated for the detection of local recurrent prostate cancer. The reported sensitivities and specificities ranged between 71 – 88%, and 94 – 100%, respectively [7,8,18]. Nevertheless, since endorectal coil placement does not allow image fusion with RT-planning CT, we assessed the sensitivity and specificity of DCE-MRI without EC for the detection of local recurrent prostate cancer.

In this study the pre-RT PSA level of the 22 patients with positive DCE-MRI findings averaged 0.74±0.64 ng/mL (median, 0.51 ng/mL, range, 0.11-2.38) and 0.57±0.58 ng/ml (median, 0.34 ng/mL; range, 0.08-2.38 ng/mL) for the entire group. Against the background of lower pre-RT PSA levels compared to previous studies that investigated the sensitivity of DCE-MRI with EC in the detection of PC recurrence (PSA, averaged 0.8 - 1.26 ng/mL), the sensitivity of 67% reported in this study appears to be comparable to the performance of DCE-MRI with EC (sensitivity, 71 – 88%) [7,8,18].

In the normal post-surgical anatomy of the male pelvis following radical prostatectomy variable degrees of post-surgical fibrosis may be present [19,20]. However, no enhancement of the prostatic bed in the arterial phase after administration of i.v. gadolinium had been described by Allen et al. [17] or had been found in the control group of Sciarra et al. [8]. In addition to these results we describe a frequent contrast enhancement inferior of the vesicourethral anastomosis, anterior to the proximal urethra in 27 of the 33 post-RP patients (82%) with distinctive morphologic characteristics as described above (Figure 4a and b). This finding would be familiar to radiologists with thorough experience of prostate- and urogenital MRI-imaging representing physiological periurethral vascular elements in the region of the diaphragma urogenitale, which can be seen regularly in contrast enhanced MRI of the male pelvis without prior surgery [17,21,22]. Six of the 33 patients (18%) did not exhibit these characteristic changes. This might be explained by varying resection margins during RP. To avoid false positive readings knowledge of this non-disease-specific structure is crucial for correct image interpretation. Local recurrent tumor nodules tend to be located most frequently around the posterior and lateral aspects of the vesico-urethral anastomosis and rather rarely in the anterior aspects as we found in our cohort which is in concordance with previous studies [15].

### Limitations of the study

First, this is a retrospective study and the results need to be confirmed in future prospective clinical trials.

Second, histopathologic confirmation of the DCE-MRI findings was not available. However, detection rates using ultrasound or MRI guided biopsy are reported to be low (range, 30 – 66%), with a positive biopsy of less than 30% in patients with PSA levels <1 ng/mL [5,8,23-25].

We used the post-RT DCE-MRI (which was performed at median 15 months after salvage RT) and complete PSA response as a negative control. This is justifiable, since the combination of a negative post-RT DCE-MRI and unremarkable PSA levels rule out macroscopic disease. Whether the high true negative rate reported in this study stands up to a control group of post-RP patients without local recurrent PC needs further investigation, however, our high true negative rate confirms reports of other authors [8,17].

## Conclusions

DCE-MRI without endorectal coil may detect local recurrent PC at low PSA levels with acceptable accuracy. All false negative DCE-MRI scans were detected using a PSA cut-off of ≥0.54 ng/mL. This image modality may therefore be considered in radiation oncology as a tool to more accurately define the gross tumor volume to increase the efficacy of salvage radiation treatment and to decrease radiation side effects.

## Competing interests

The authors' declare that they have no competing interests.

## Authors’ contributions

HCR carried out imaging analyses, the drafting of the manuscript and partially the analysis and interpretation of the data. AOS carried out the design of the MRI scans. HCR and UN carried out the design of the study. NV and KH participated in the analysis of the data. MRB participated in the analysis and statistics of the data. WSS, ML and ALG participated in the methodological design. All authors read and approved the final manuscript.
